# Autophagic blockade potentiates anlotinib-mediated ferroptosis in anaplastic thyroid cancer

**DOI:** 10.1530/ERC-23-0036

**Published:** 2023-08-01

**Authors:** Jiajun Wu, Juyong Liang, Ruiqi Liu, Tian Lv, Kangyin Fu, Liehao Jiang, Wenli Ma, Yan Pan, Zhuo Tan, Qing Liu, Weihua Qiu, Minghua Ge, Jiafeng Wang

**Affiliations:** 1 Graduate Department, Bengbu Medical College, Bengbu, Anhui, China; 2 Otolaryngology & Head and Neck Center, Cancer Center, Department of Head and Neck Surgery, Zhejiang Provincial People’s Hospital (Affiliated People’s Hospital), Hangzhou Medical College, Hangzhou, Zhejiang, People’s Republic of China; 3 Key Laboratory of Endocrine Gland Diseases of Zhejiang Province, Hangzhou, Zhejiang, People’s Republic of China; 4 Clinical Research Center for Cancer of Zhejiang Province, Hangzhou, Zhejiang, People’s Republic of China; 5 Department of Thyroid and Breast Surgery, Zhejiang Provincial People’s Hospital Bijie Hospital, Bijie, Guizhou, China; 6 Department of General Surgery, Ruijin Hospital, Shanghai Jiao Tong University School of Medicine, Shanghai, China

**Keywords:** anaplastic thyroid cancer (ATC), anlotinib, ferroptosis, autophagy

## Abstract

Anlotinib-mediated angiogenic remodeling was delineated in various tumors. Meanwhile, we previously showed that anlotinib inhibited tumor angiogenesis in anaplastic thyroid cancer (ATC). However, the potential role of anlotinib on cell lethality in ATC remains an enigma. Herein, we found that anlotinib inhibited the viability, proliferation, and migration of KHM-5M, C643, and 8505C cells in a dose-dependently manner. Under anlotinib treatment, PANoptosis (pyroptosis, apoptosis, and necroptosis) markers were not changed; however, ferroptosis targets (transferrin, HO-1, FTH1, FTL, and GPX4) were significantly downregulated. ROS levels also increased in a concentration-dependent manner after anlotinib treatment in KHM-5M, C643, and 8505C cells. In addition, protective autophagy was activated in response to anlotinib, and autophagic blockade potentiated anlotinib-mediated ferroptosis and antitumor effects *in vitro* and *in vivo*. Our new discovery identified autophagy-ferroptosis signaling pathway which provides mechanistic insight into anlotinib-mediated cell death, and synergistic combination therapy may help develop new ATC treatment strategies.

## Introduction

**Figure 1:**
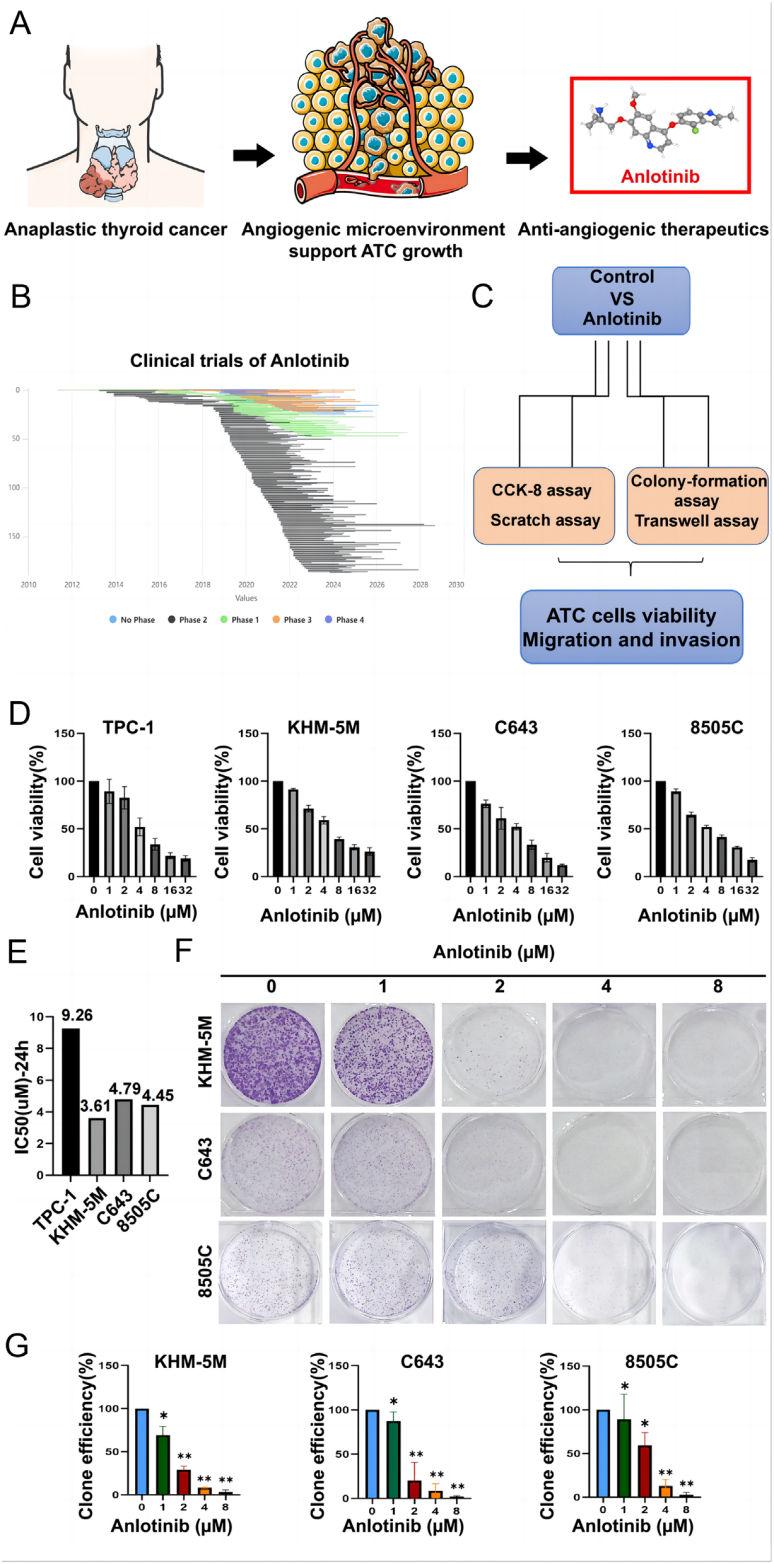
Anlotinib inhibited the proliferation of ATC cells. (A) Anti-angiogenic therapeutics is the mainstay treatment for ATC. (B) The clinical trials of anlotinib in multiple tumors. (C) Flowchart of anlotinib suppressing the malignant behaviors of ATC cells. (D) ATC cells (TPC-1, KHM-5M, C643, and 8505C) were treated with control medium or a series of concentrations of anlotinib (0, 1, 2, 4, 8, 16, and 32 µM) for 24 h. Cell viability was assessed by CCK-8 assay. (E) The IC-50 value of anlotinib treatment in TPC-1, KHM-5M, C643, and 8505C cells for 24 h. (F) and (G) Representative images of contact-dependent clone formation and quantification of the clone number in KHM-5M, C643, and 8505C cells. Each experiment was representative of three independent experiments. All data were obtained from three independent experiments. *P < 0.05; **P < 0.01.

Thyroid cancer is one of the most common endocrine malignancies with increasing incidence (Cabanillas *et al.* 2016, Sung *et al.* 2021). Although ATC accounts for only 2% of all thyroid cancers, it is responsible for the majority of thyroid cancer deaths due to its high aggressiveness and few available treatment options (Perrier *et al.* 2018, Bible *et al.* 2021). Considering the functional role of neovascularization in ATC progression, antiangiogenic therapy has become a mainstay treatment for ATC suppression (Fig. 1A and B, Zhu *et al.* 2010, Ravaud *et al.* 2017, Feng *et al.* 2021).

Anlotinib, a multitarget receptor tyrosine kinase inhibitor, has already achieved remarkable efficacy in lung cancer and osteosarcoma due to its potent antiangiogenic properties (Han *et al.* 2018, Wang *et al.* 2019, Su *et al.* 2022). In addition, our previous study confirmed that anlotinib could suppress ATC carcinogenesis by inhibiting angiogenesis *in vitro* and *in vivo* (Liang *et al.* 2021). However, the potential role of anlotinib on cell lethality in ATC remains unclear.

Ferroptosis is a novel nonapoptotic regulated cell death process (Jiang* et al.* 2021*b*, Tang *et al.* 2021). It is characterized by abnormal cysteine and glutathione metabolism and iron-dependent accumulation of peroxidized lipids. Accumulating evidence suggests the importance of pro-ferroptosis strategies in cancer treatment, especially in drug-resistant malignancies (Hassannia *et al.* 2019, Chen *et al.* 2021). This study aims to investigate the regulatory mechanism underlying anlotinib-mediated ferroptosis and identify potential combination therapies for ATC.

## Materials and methods

### Cell culture and reagents

Human ATC cell lines KHM-5M, C643, and 8505C and PTC cell line TPC-1 were conserved by the Institute of Clinical Medicine, Zhejiang Provincial People’s Hospital. Cell lines were cultured in RPMI-1640 (Hyclone, China) with 10% fetal bovine serum (KEL Biotech, Shanghai, China). Cells were cultured at 37°C in 5% CO_2_. All cells were preserved at −80°C using CELLSAVING (New Cell & Molecular Biotech, Suzhou, Jiangsu, China). Anlotinib (AL3818) and chloroquine (T0194) were, respectively, purchased from Shanghai Lanmu Chemical Co. (Shanghai, China) and Shanghai Taoshu Biotechnology Co. (Shanghai, China), and were dissolved in sterile purified water and diluted with medium to the desired concentration. Recombinant human CXCL11 (rhCXCL11) and recombinant human HB-EGF (rhEGF) were purchased from PeproTech (Cranbury, NJ, USA).

### CCK-8 assay

The cytotoxicity of anlotinib was assessed by the CCK-8 (Beyotime Biotechnology, Shanghai, China) method. ATC cells (4000 per well) were seeded in 96-well plates and were treated with anlotinib at 0, 1, 2, 4, 8, 16, and 32 µM for 24 h. At the test point, 100 μL CCK-8 was added and then the viability values were detected by spectrometer (BioTek).

### Colony-formation assay

ATC cells (1000 per well) were seeded in six-well plates and incubated at 37°C in 5% CO_2_ for 24 h. The six-well plates were washed three times with sterile PBS to remove the exfoliated cells. Then, 0, 1, 2, 4, and 8 µM of anlotinib were added. One week later, 500 µL paraformaldehyde was added to each well for 30-min fixation, and then the fixative was discarded and replaced by 500 μL of crystalline violet dye per well. After 30-min staining, the clone number could be calculated.

### Scratch assay

ATC cells (300,000 per well) were seeded in 12-well plates for 48 h. After scratching with a gun tip in the center of the adherent cells, the 12-well plates were rinsed three times with sterile PBS. Then, 0, 1, 2, 4, and 8 µM anlotinib diluted with serum-free RPMI-1640 was added. The migration ability was assessed under a light microscope at 40× and 100× for 0, 6, 12, and 24 h.

### Transwell assay

Migration and invasion assay were performed using Transwell Permeable Plate (LABSELECT, Hefei, Anhui, China, 6.5 mm). A serum-free medium (200 μL) containing 5 × 10^4^ ATC cells was added to the upper chamber, and 700 µL of a series of concentrations of anlotinib (0, 1, 2, 4, and 8 µM) diluted with serum-containing medium were added to the lower chamber. After incubation of 24 or 48 h, cells were stained with 0.1% crystal violet for 30 min. The number of migrating cells was normalized to the number of total cells and was calculated per microscopic field. The mean number was estimated by counting average cells in five visual fields of three independent experiments.

### Western blot analysis

Western blot (WB) was performed as previously described (Jin *et al.* 2020). All protein samples were lysed in WB and IP (immunoprecipitation) cell lysate and then quantified using the BCA Protein Analysis Kit (Thermo Scientific). Proteins were separated by 15–20% SDS-PAGE gel and transferred onto PVDF membranes. After the block with 5% skimmed milk prepared of 20% TBST for 2 h, membranes were incubated at 4°C overnight with primary antibodies. Blots were probed with rabbit anti-GSDMD (ab209845 1:1000), rabbit anti-caspase 1 (24232T 1:1000), rabbit anti-cleaved-caspase 1 (4199T 1;1000), rabbit anti-cleaved GSDMD (36425T 1:1000), rabbit anti-GSDME (ab215191 1:3000), rabbit anti-cleaved GSDME (ab215191 1:3000), rabbit anti-PARP (9532T 1:1000), rabbit anti-caspase 3 (ab184787 1:2000), rabbit anti-cleaved-PARP (5625T 1:2000), rabbit anti-cleaved-caspase 3 (9661T 1:1000), mouse anti-caspase 8 (9746T 1:5000), rabbit anti-RIP (3493T 1:1000), rabbit anti-P-RIP(65746T 1:1000), mouse anti-cleaved-caspase 8 (9746T 1:5000), rabbit, anti-ATG7 (ab52472 1:1000) , rabbit anti-Beclin1 (ab210498 1:1000) , rabbit anti-LC3B (ab192890 1:2000), rabbit anti-P62 (ab207350 1:1000), rabbit anti-transferrin (TFR, A9130 1: 5000), rabbit anti-HO-1 (10701-1-AP 1:2000), rabbit anti-GPX4 (ab125066 1:5000), rabbit anti-FTH1 (A19544 1:5000), rabbit anti-FTL (A11241 1:5000), and mouse anti-GAPDH (ab8245 1:2000). Goat anti-rabbit or anti-mouse horseradish peroxidase-conjugated IgG was used as secondary antibody (Santa Cruz Biotechnology). Finally, the protein bands were analyzed using chemiluminescent substrate HRP (Verde Biotechnology, Hangzhou, Zhejiang, China).

### Flow cytometry analysis

Flow cytometry was performed as previously described (Feng *et al.* 2018). Cells were treated with 0, 1, 2, 4, 8, 16, and 32 µM anlotinib for 8 h and then were incubated with an H2DCFDA probe for 30 min under a dark environment. The residual dye was washed with ice-cold PBS, and suspended in 100 µL serum-free medium. Final measurements were performed on a flow cytometer (Beckman Coulter Ireland Inc.). The fluorescence of each probe was measured using the FlowJo software program.

### Immunofluorescence

Immunofluorescence (IF) was performed as previously described (Feng *et al.* 2022). Cells were treated with 0, 1, 2, 4, 8, 16, and 32 µM anlotinib for 8 h and then were incubated with DCFA and Hoechst probes for 30 min and 10 min under a dark environment. Finally, images were collected by using a confocal microscope.

### *In vivo* xenograft tumor model and immunohistochemistry

ATC xenograft models in nude mice were established (Liang *et al.* 2021). Three-week-old female BALB/c nude mice were purchased from Shanghai SLAC Laboratory Animal Co. Ltd. (Shanghai, China). All experiments were performed following the official recommendations of the Chinese Society of Zoology, and animals received humane care according to the standards listed in the Ethical Review Form for Laboratory Animal Welfare. Suspensions containing 8505C cell were subcutaneously injected into the right flank of the nude mice. After approximately 2 weeks, when the tumor diameter reached approximately 5 mm, all mice were randomly categorized into four different groups, including control, anlotinib (3 mg/kg), chloroquine (60 mg/kg), and combined treatment with anlotinib and chloroquine groups (five mice in each group). Anlotinib and chloroquine were administered by oral gavage and intraperitoneal injection, respectively. Tumor size and volume were recorded every 2 days. Tumor size was measured using vernier calipers and tumor volume was calculated using the following formula: *V* = *W*^2^ × *L*/0.5. Three days after the last injection, animals were executed by CO_2_ inhalation, and tumors were removed, weighed, and fixed in formalin. Immunohistochemistry was performed as previously described (Feng *et al.* 2021). Immunohistochemistry images were evaluated by the pathologist. Immunohistochemistry scoring was completed according to the percentage of positive cells (0 = 0–5%, 1 = 5–25%, 2 = 26–50%, 3 = 51–75%, 4 = 76–100%) and the staining intensity (0 = negative, 1 = weak, 2 = moderate, 3 = strong). The two scores were multiplied to generate an immunoreactive score ranging from 0 to 12 (Liang *et al.* 2021).

### Statistics

Statistical analyses were processed using GraphPad Prism 8.0. One-way ANOVA and the Student’s *t*-test were chosen for comparison among groups. Categorical data were evaluated with the chi-square test or Fisher’s exact test. *P* < 0.05 were considered significant.

## Results

### Anlotinib suppressed the malignant behaviors of ATC cells

TPC-1, KHM-5M, C643, and 8505C cells were incubated with a series of concentrations of anlotinib for 24 h (Fig. 1C). The CCK8 results showed that anlotinib hardly decreased cell viability at concentrations less than 1 μM, and cell death was significantly induced at concentrations greater than 4 µM (Fig. 1E). The IC-50 values of TPC-1, KHM-5M, C643, and 8505C were 9.49, 6.01, 4.85, and 4.46 µM, respectively (Fig. 1D). To evaluate the inhibitory effect of anlotinib on the proliferation of ATC cells, we found that the number of clones of KHM-5M, C643, and 8505C cells decreased in a dose-dependent manner by using a colony assay. Moreover, the number of cell clones was markedly reduced at concentrations greater than 2 μM (Fig. 1F and G). These* in vitro* results indicated the inhibitory effect of anlotinib on ATC cell viability.

**Figure 2:**
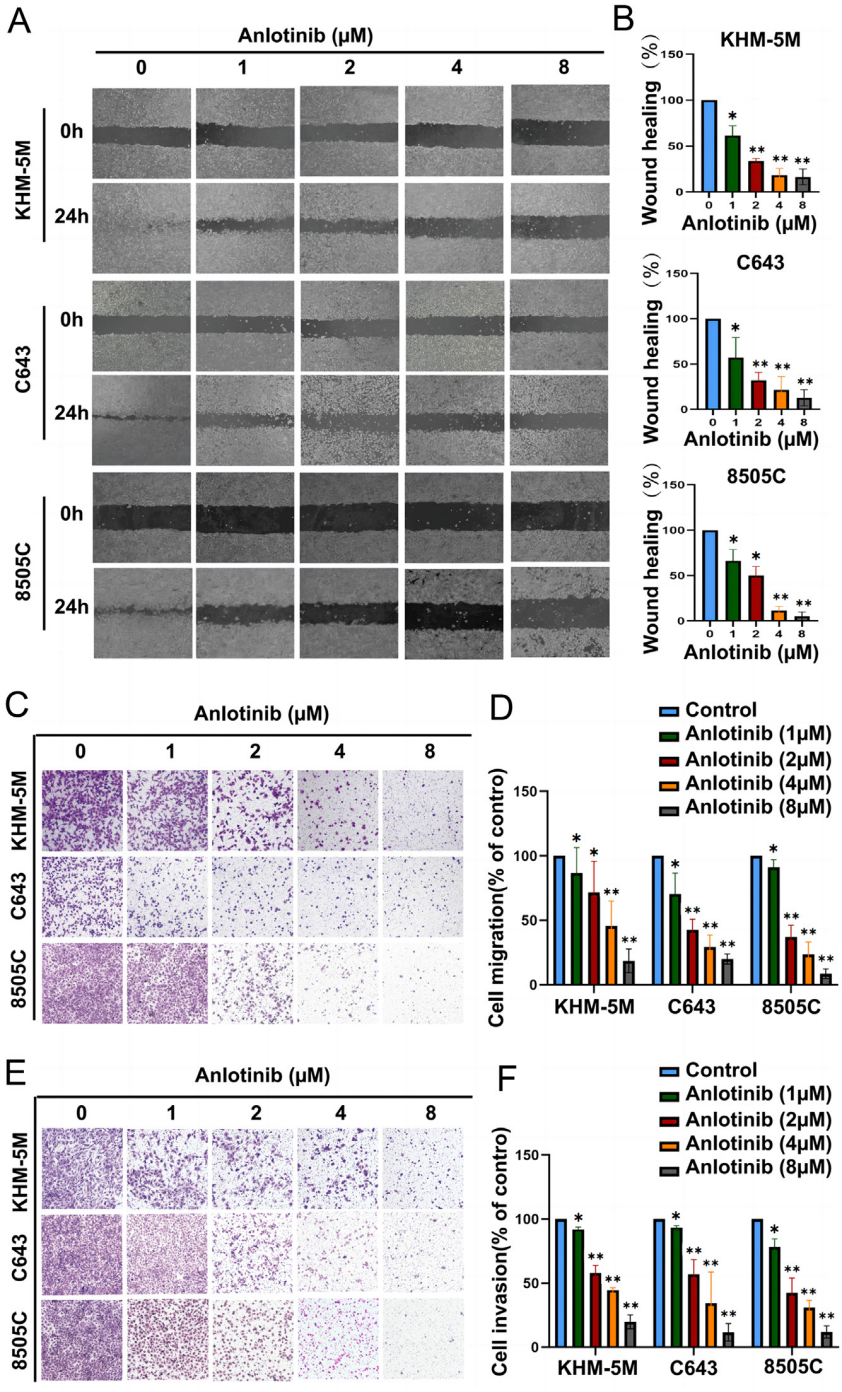
Anlotinib suppressed ATC migration and invasion abilities. (A) and (C) Compared with the control groups, anlotinib decreased the migration ability of KHM-5M, C643, and 8505C cells in a dose-dependent manner. (B) and (D) Histograms show the relative wound healing area and migration number of cells. (E) and (F) The invasion ability of KHM-5M, C643, and 8505C cells was inhibited upon anlotinib treatment. Histograms show the invasion number of cells. All data are obtained from three independent experiments. *P < 0.05; **P < 0.01.

Scratch and Transwell assays were employed to investigate whether anlotinib could affect the migration and invasion ability of ATC cells. A wider wound healing area and fewer migrating cells were observed in the anlotinib group. When the concentration of anlotinib reached 4 μM, the migration ability of ATC cells was significantly weakened (Fig. 2A, B, C and D). Furthermore, the results of the Transwell invasion assay indicated that anlotinib could reduce the number of ATC cells in the lower chamber (Fig. 2E and F). In order to exclude the impact of EGF and CXCL11 on anlotinib-induced anti-migrating ability (Liang *et al.* 2021), we added rhCXCL11 and rhEGF to tumor cell with or without anlotinib treatment and found that invasion and migration of ATC cells could hardly be influenced (Supplementary Fig. 1A, B, C and D). Therefore, the anlotinib-mediated malignant suppression is angiogenesis independent. Altogether, the migration and invasion ability of ATCs was inhibited upon anlotinib treatment.

### Anlotinib-mediated cell death occurred mainly via ferroptosis but not PANoptosis

**Figure 3:**
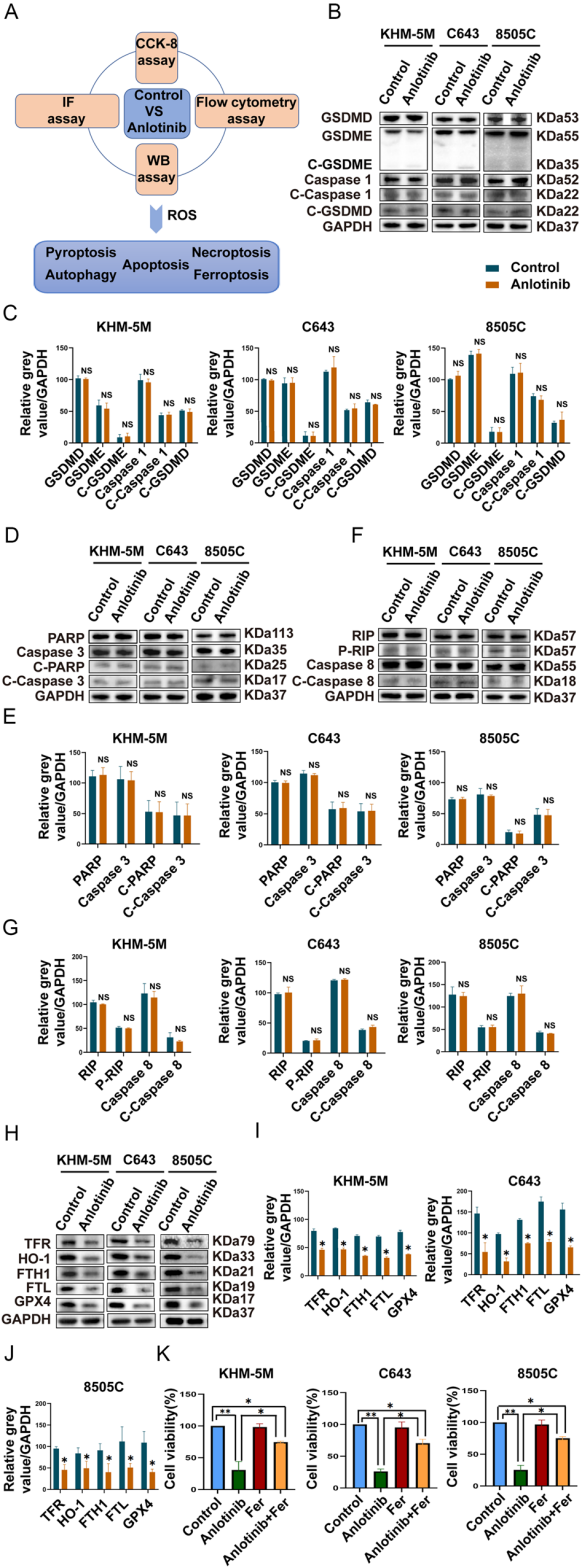
Anlotinib-mediated cell death occurred mainly *via* ferroptosis but not PANoptosis. (A) Flowchart of anlotinib-mediated cell death in ATC cells. (B)–(G) KHM-5M, C643, and 8505C cells were treated with anlotinib for 24 h. PANoptosis marker expression (pyroptosis (GSDMD, GSDME, C-GSDME, Caspase 1, C-Caspase 1, and C-GSDMD), apoptosis (PARP, Caspase 3, C-PARP, and C-Caspase 3), and necroptosis (Caspase 8, C-Caspase 8, RIP, and P-RIP) were detected by western blot. (H)–(J) Ferroptosis markers (GPX4, FTH1, FTL, transferrin, and HO-1) were detected by western blotting. (K) Compared with the anlotinib groups, the cell viability was reversed when ATC cells were coincubated with anlotinib and ferroptosis inhibitor (FER-1). All data are obtained from three independent experiments. *P < 0.05; **P < 0.01.

PANoptosis emphasizes the signal crosstalk between various regulated cell death types, including pyroptosis, apoptosis, and necroptosis. To identify the precise pathway of cell death under anlotinib treatment, PANoptosis markers were examined by western blotting (Fig. 3A). Interestingly, the markers of pyroptosis (GSDMD, GSDME, C-GSDME, Caspase 1, C-Caspase 1, and C-GSDMD), apoptosis (PARP, Caspase 3, C-PARP, and C-Caspase 3), and necroptosis (Caspase 8, C-Caspase 8, RIP, P-RIP) were not significantly altered after anlotinib intervention (Fig. 3B, C, D, E, F and G). Therefore, anlotinib-mediated ATC cell death may not occur through PANoptosis.

Then, we further explored whether anlotinib could trigger metabolic dysfunction and ferroptosis. Consequently, five targets (transferrin, HO-1, FTH1, FTL, and GPX4) were significantly downregulated after treatment with anlotinib compared with the control group in KHM-5M, C643, and 8505C cells (Fig. 3H, I and J). In addition, after ATC cells were coincubated with anlotinib and a ferroptosis inhibitor (FER-1), the suppressed cell viability was reversed by FER-1 (Fig. 3K).

**Figure 4:**
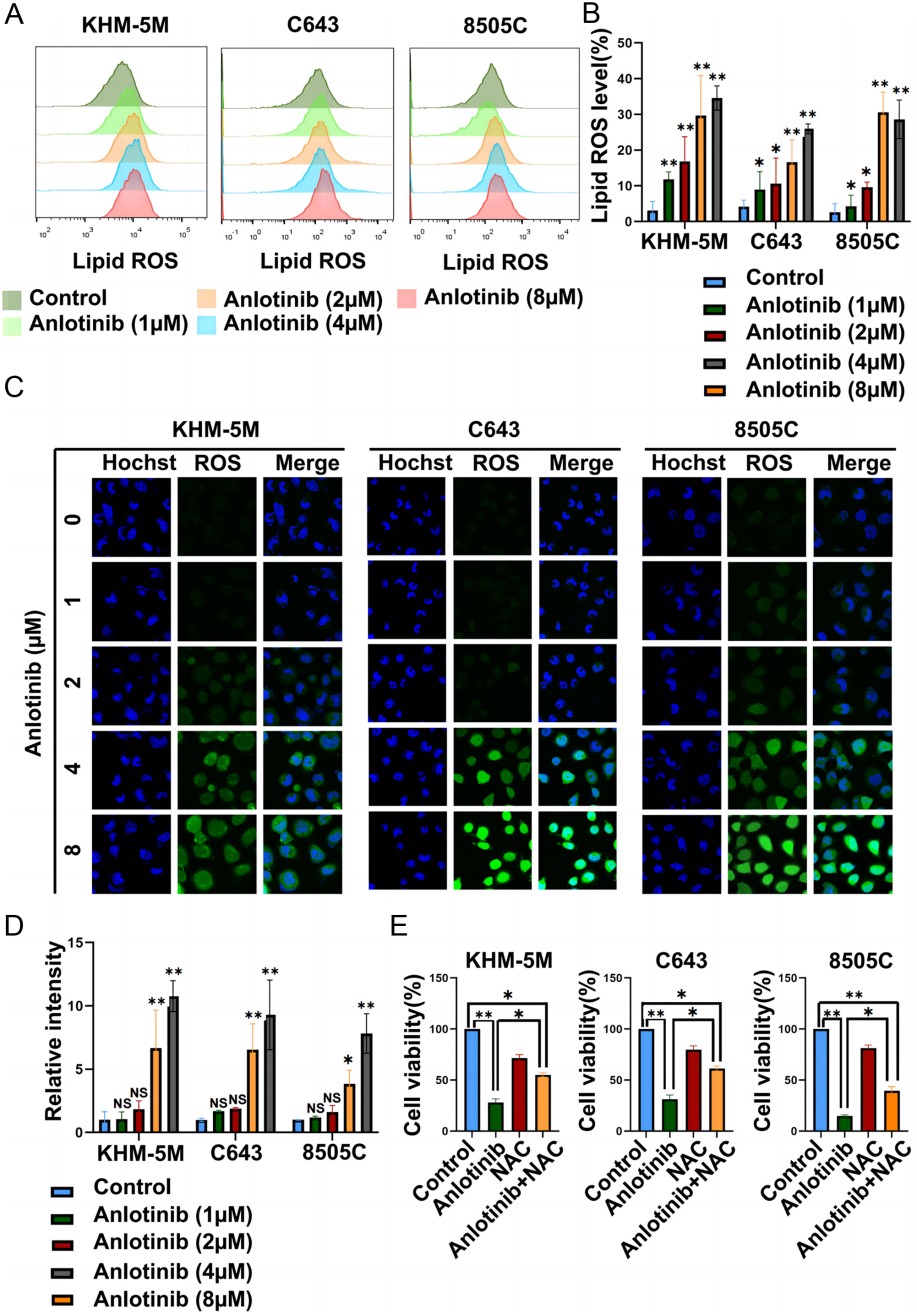
Anlotinib increased ROS levels in ATC cells. (A)–(D) The levels of lipid ROS were detected under anlotinib treatment in KHM-5M, C643, and 8505C cells with an H2DCFDA probe viaflow cytometry and immunofluorescence. (E) Compared with the anlotinib groups, the cell viability was reversed by NAC when ATC cells were coincubated with anlotinib and ROS inhibitor (NAC). All data are obtained from three independent experiments. *P < 0.05; **P < 0.01.

Considering that ROS play a central role during ferroptosis, we hypothesized that anlotinib could induce an ROS homeostasis disorder in ATC. By using flow cytometry, ROS levels increased in a concentration-dependent manner after anlotinib treatment in KHM-5M, C643, and 8505C cells (Fig. 4A and B). Furthermore, ROS changes were visualized by IF. Compared with the control group, more ROS signals were detected in the anlotinib group (Fig. 4C and D). Then, we tried to reverse ROS with the ROS scavenger NAC, and we found that ATC cell viability in the anlotinib + NAC group was significantly higher than that in the anlotinib-alone group (Fig. 4E). We further investigated the potential effect of EGF and CXCL11 on ferroptosis (Liang *et al.* 2021). We added rhCXCL11 and rhEGF to tumor cell with or without anlotinib treatment and found that anlotinib mediated ferroptosis effect is CXCL11 and EGFR independent (Supplementary Fig. 1I, J, K and L). Altogether, these results preliminarily indicated that anlotinib elicited antitumor effects *via* ferroptosis in ATC and that ROS were dysregulated.

### Autophagic blockade potentiated anlotinib-mediated ferroptosis

**Figure 5:**
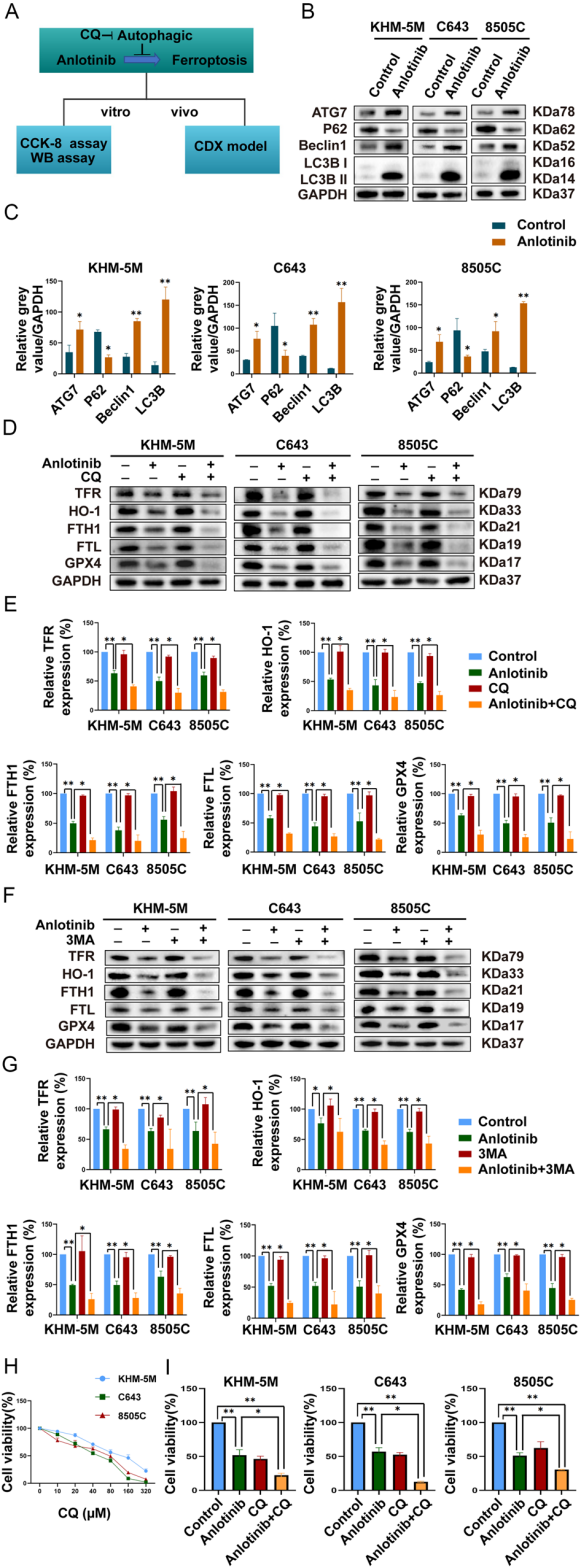
Autophagic blockade potentiates anlotinib-mediated ferroptosis. (A) Flowchart investigating the crosstalk between autophagy and ferroptosis under anlotinib treatment. (B) and (C) KHM-5M, C643, and 8505C cells were treated with a series of concentrations of anlotinib for 8 h. The expression levels of autophagy markers (ATG7, P62, Beclin 1, LC3-II) and GAPDH were detected by western blot. (D)–(G) KHM-5M, C643, and 8505C cells were treated with anlotinib with or without autophagy inhibitor (CQ 100 µM, or 3MA 10 mM) for 24 h. GPX4, FTH1, FTL, transferrin, and HO-1 were detected by WB. (H) The CCK8 results of ATC cells treated with the control medium or a series of concentrations of CQ (0, 10, 20, 40, 80, 160, and 320 µM) for 24 h. (I) Compared with the anlotinib group, cell lethality was potentiated when ATC cells were coincubated with anlotinib and CQ. All data are obtained from three independent experiments. *P < 0.05; **P < 0.01.

Accumulating studies have shown that autophagy functions importantly during ferroptosis (Dai *et al.* 2020, Wei *et al.* 2020). Therefore, autophagic markers were examined in our series (Fig. 5A). In KHM-5M, C643, and 8505C cells, the expression of P62 was significantly reduced in response to anlotinib, while the increased expression of LC3B-II, ATG7, and Beclin1 further validated the autophagic effect of anlotinib (Fig. 5B and C).

To further explore whether autophagy played a protective role in anlotinib-mediated ferroptosis, we initially confirmed that autophagy inhibitor (CQ or 3MA) alone could hardly alter the baseline level of ferroptosis under a series of concentrations (Supplementary Fig. 1E, F, G and H). Then, we divided ATC cells into four groups (control group, anlotinib group, autophagy inhibitor group (CQ or 3MA), and anlotinib + autophagy inhibitor group). Consequently, CQ and 3MA hardly influenced the expression level of ferroptosis markers; however, adding CQ and 3MA to anlotinib resulted in lower levels of ferroptosis markers (GPX4, FTH1, FTL, transferrin, and HO-1) than anlotinib treatment alone (Fig. 5D, E, F and G). In addition, the CCK8 results further validated that inhibition of autophagy could enhance anlotinib-induced ferroptosis in KHM-5M, C643, and 8505C cells (Fig. 5H and I).

### CQ improved anlotinib-mediated antitumorigenesis *in vivo*

**Figure 6:**
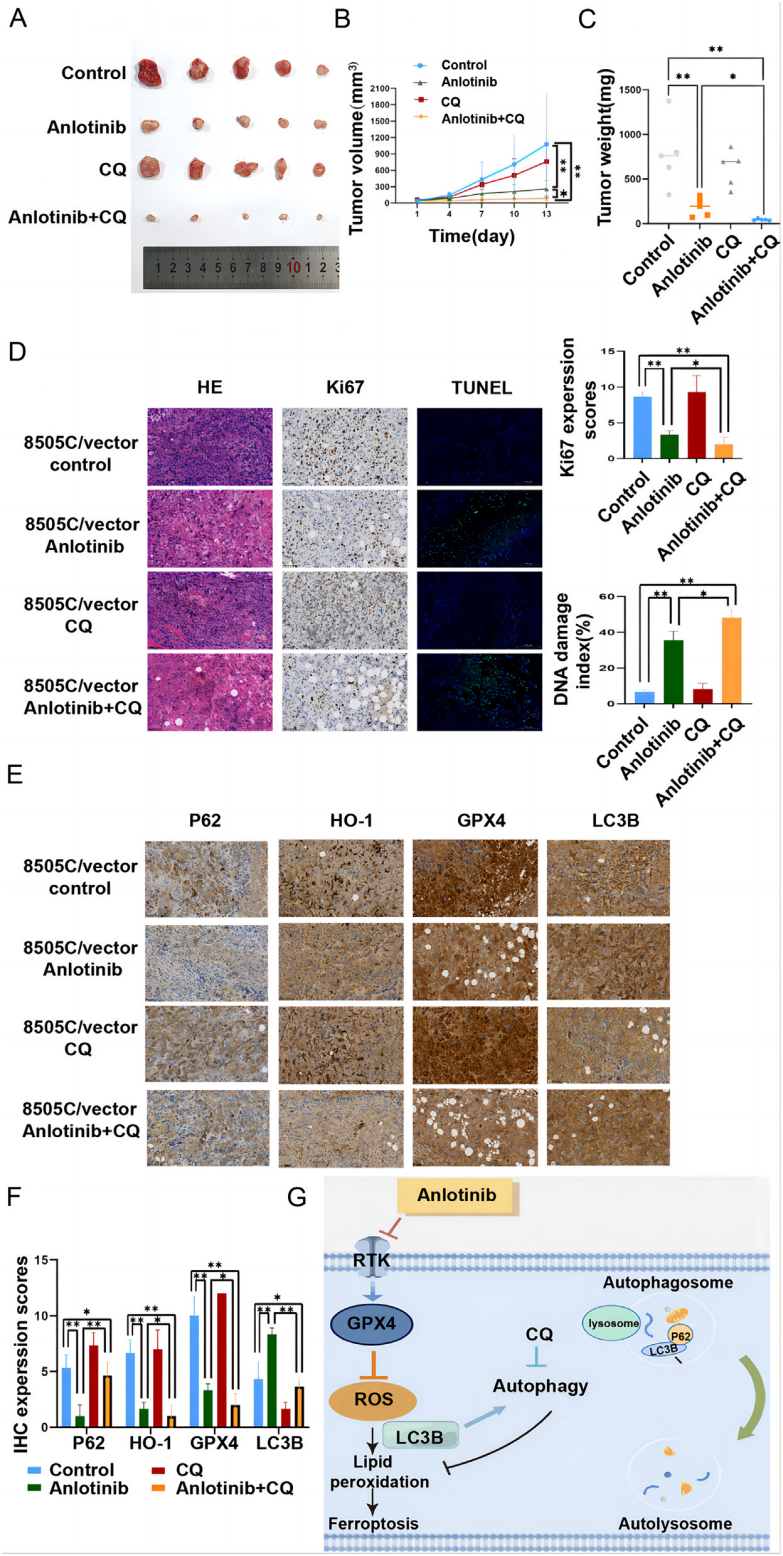
CQ improved anlotinib-mediated antitumorigenesis *in vivo*. (A) Xenograft models were generated by injecting 8505C. Four groups of mice were treated with the control medium, anlotinib, CQ, and a combination of two agents. (B) and (C) Quantification of tumor volumes and weights of the four groups. (D) and (E) Images and quantifications of TUNEL assay and IHC staining of Ki67, P62, LC3B, HO-1, and GPX4. (F) Schematic of anlotinib-mediated autophagy-ferroptosis signaling in anaplastic thyroid cancer.

Considering the *in vitro* findings, a xenograft model was used to validate the protective effect of autophagy *in vivo*. Mice with ATC tumors were exposed to anlotinib, CQ, and combination treatment groups. Comparison with the control group indicated that treatment with anlotinib decreased tumor volume, and the tumor volumes in the CQ treatment group did not show a significant difference compared with those in the control group. Moreover, the combination of CQ with anlotinib significantly enhanced the inhibitory effect compared with the anlotinib group (Fig. 6A and B). Consistent with the results of tumor volume, tumor weight could be suppressed by anlotinib, and a combination of CQ with anlotinib showed a more impressive antitumorigenesis effect (Fig. 6C).

Tumorigenic markers were assayed by IHC. A comparison with the control group indicated that the tumors in the anlotinib group had lower expression levels of Ki67 and ferroptosis molecules. Enhanced TUNEL staining was observed in the anlotinib group (Fig. 6D, E and F).

## Discussion

ATC is a highly aggressive malignancy with treatment resistance (Wendler *et al.* 2016, Prasongsook *et al.* 2017). Despite comprehensive treatment, the prognosis of ATC patients has not improved significantly (Dierks *et al.* 2021, Liang *et al.* 2022). Anlotinib has been seen as a potent angiogenic modulator for treating ATC (Liang *et al.* 2021). However, this study was the first to illuminate the potential regulatory role of anlotinib in ferroptosis.

At present, accumulating studies argue for complex crosstalk between various cell death types, such as pyroptosis, apoptosis and necroptosis (Bertheloot *et al.* 2021). These regulated cell death types have gradually led to the concept of PANoptosis, which is triggered by the formation of the inflammatory PANoptosis complex (Zheng & Kanneganti 2020, Lee *et al.* 2021). In addition, the activation of caspase 8 is the key regulator of the inflammatory response and the focal point of converging pathways that mediate PANoptosis (Kesavardhana *et al.* 2020, Jiang* et al.* 2021*a*). Previous studies have shown that anlotinib induces cell lethality mainly through PANoptosis (Ruan *et al.* 2019). However, in our study, the cellular markers and features of PANoptosis were not identified, and c-caspase 8 was not observed. We found that ferroptosis was significantly triggered in an ROS pathway-dependent manner upon anlotinib treatment in ATC. In contrast to the inflammatory process in PANoptosis, ferroptosis is characterized by metabolic dysfunction and iron-dependent accumulation of peroxidized lipids. Considering the resistance to apoptotic inducers in various tumors, killing tumor cells by promoting ferroptosis may become a potential gateway to overcome multiple drug resistance in the anticancer therapy (Juchum *et al.* 2015, Liang *et al.* 2019). Therefore, illuminating the regulatory mechanism of ferroptosis in ATC may further broaden the therapeutic potential of anlotinib.

During anlotinib-mediated ferroptosis, autophagy was enhanced and exerted a protective impact on cell viability. However, autophagy inhibitors alone can hardly promote ferroptosis, partially because the level of protective autophagy cannot be activated without anlotinib treatment. The interaction between autophagy and ferroptosis has been partly investigated previously (Dai *et al.* 2020). Specifically, autophagy leads to the degradation of cellular ferritin; thus, the balance of intracellular iron is destroyed (Gupta *et al.* 2023). The existence of intracellular free iron can increase the concentration of reactive oxygen species, which triggers ferroptosis. In this study, protective autophagy was observed, and autophagic blockade potentiated anlotinib-mediated ferroptosis and antitumor effects.

In summary, this study is the first to demonstrate the effects of anlotinib on ferroptosis. The identified autophagy-ferroptosis signaling pathway may provide a potential combination therapeutic strategy for ATC.

## Supplementary Materials

Supplemental Figure 1 A-D The impact of rhCXCL11 (100ng/ml) and rhEGF (10nmol/L) with or without anlotinib on invasion and migration were investigated in KHM-5M, C643 and 8505C cells. Histograms show the migration number and invasion number of cells. E-H KHM-5M, C643 and 8505C cells were treated with control medium or a series of concentrations of CQ (0, 50 and 100µM) or 3MA (0, 5 and 10mM) for 24 h. GPX4, FTH1, FTL, transterrin, and HO-1 were detected by WB. I-L KHM-5M, C643 and 8505C cells were treated with anlotinib with or without rhCXCL11 and rhEGF for 24 h. The expression levels of ferroptosis markers (GPX4, FTH1, FTL, transterrin, and HO-1) and GAPDH were detected by western blot. All data are obtained from three independent experiments. *P < 0.05; **P < 0.01.

## Declaration of interest

The authors declare that there is no conflict of interest that could be perceived as prejudicing the impartiality of the research reported.

## Funding

This study was supported in part by grants from the Basic Public Welfare Research Program Foundation of Zhejiang Province (Grant number: LGF22H160049, to Jiafeng Wang); Medical and Health Science Research Foundation of Zhejiang Province (Grant number: 2021KY055, to Jiafeng Wang); Zhejiang Provincial Natural Science Foundation of China under Grant No. LY22H160036 (to Zhuo Tan); Zhejiang Provincial Natural Science Foundation of China under Grant No. LQ23H160050 (to Juyong Liang); Nature Science Foundation of China (Grant number: 82103027, to Tian Lv); Zhejiang Provincial Natural Science Foundation of China under Grant No. LY23H160025 (to LieHao Jiang); Zhejiang Health Science and Technology Project (Grant number: 2022KY525, to LieHao Jiang).

## Ethical committee approval

The animal experiments involved in this study were approved by the Laboratory Animal Management and Ethics Committee of Zhejiang Provincial People's Hospital (Approval number: IACUC-A20220023).

## Author contribution statement

JF.W. and MH.G. designed the study. JY.L. analyzed the data and revised the manuscript. JJ.W. wrote the manuscript and performed most of the experiments. RQ.L. and WL.M. carried out data curation and visualization. T.L., Y.P., and KY. F. carried out investigation. LH.J. and Z.T. carried out supervision and project administration. Q.L. and WH.Q. designed the revision and performed the part of experiments. All of the authors discussed the results, reviewed, and approved the final manuscript.
