# ERRATUM: Autophagic blockade potentiates anlotinib-mediated ferroptosis in anaplastic thyroid cancer

**DOI:** 10.1530/ERC-23-0036e

**Published:** 2025-01-10

**Authors:** Jiajun Wu, Juyong Liang, Ruiqi Liu, Tian Lv, Kangyin Fu, Liehao Jiang, Wenli Ma, Yan Pan, Zhuo Tan, Qing Liu, Weihua Qiu, Minghua Ge, Jiafeng Wang

**Affiliations:** ^1^ Graduate Department, Bengbu Medical College, Bengbu, Anhui, China; ^2^ Otolaryngology & Head and Neck Center, Cancer Center, Department of Head and Neck Surgery, Zhejiang Provincial People’s Hospital (Affiliated People’s Hospital), Hangzhou Medical College, Hangzhou, Zhejiang, People’s Republic of China; ^3^ Key Laboratory of Endocrine Gland Diseases of Zhejiang Province, Hangzhou, Zhejiang, People’s Republic of China; ^4^ Clinical Research Center for Cancer of Zhejiang Province, Hangzhou, Zhejiang, People’s Republic of China; ^5^ Department of Thyroid and Breast Surgery, Zhejiang Provincial People’s Hospital Bijie Hospital, Bijie, Guizhou, China; ^6^ Department of General Surgery, Ruijin Hospital, Shanghai Jiao Tong University School of Medicine, Shanghai, China

The authors and journal apologise for an error in the supplementary materials of the above paper, which appeared in volume 30 part 9, article ID e230036. The error relates to Supplementary Figure 1 (see section on Supplementary materials given at the end of this article) given with the online version of the paper, in which migration images of six groups from the 8505C cell line were accidentally replaced with those from the KHM-5M cell line during figure layout. The correct figure artwork is published with this erratum. The supplementary materials associated with the version of record have been corrected.

**Supplementary Figure 1 (**A–D) The impact of rhCXCL11 (100 ng/mL) and rhEGF (10 nmol/L) with or without anlotinib on invasion and migration was investigated in KHM-5M, C643 and 8505C cells. Histograms show the migration number and invasion number of cells. (E–H) KHM-5M, C643 and 8505C cells were treated with a control medium or a series of concentrations of CQ (0, 50 and 100 μM) or 3-MA (0, 5 and 10 mM) for 24 h. GPX4, FTH1, FTL, transferrin and HO-1 were detected by WB. (I–L) KHM-5M, C643 and 8505C cells were treated with anlotinib with or without rhCXCL11 and rhEGF for 24 h. The expression levels of ferroptosis markers (GPX4, FTH1, FTL, transferrin and HO-1) and GAPDH were detected by western blot. All data are obtained from three independent experiments. *P < 0.05; **P < 0.01.

## Supplementary materials



