# Supplementary material for: Autophagic blockade potentiates anlotinib-mediated ferroptosis in anaplastic thyroid cancer
Source: Endocr Relat Cancer. 2023 Aug 1;30(9):e230036. doi: 10.1530/ERC-23-0036 (PMC10448565; doi:10.1530/ERC-23-0036)

Supplemental Fig1

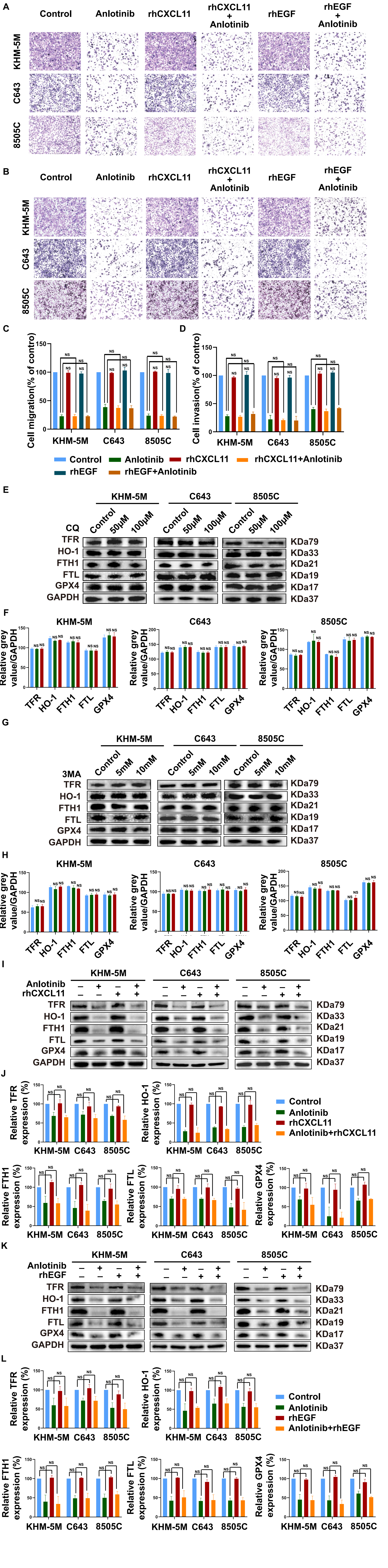

Supplement: Supplemental Figure 1 A-D The impact of rhCXCL11 (100ng/ml) and rhEGF (10nmol/L) with or without anlotinib on invasion and migration were investigated in KHM-5M, C643 and 8505C cells. Histograms show the migration number and invasion number of cells. E-H KHM-5M, C643 and 8505C cells were treated wit [file supplementary_figure_1.pdf]
